# Comparative RNA-Seq Transcriptome Analysis on Pulmonary Inflammation in a Mouse Model of Asthma–COPD Overlap Syndrome

**DOI:** 10.3389/fcell.2021.628957

**Published:** 2021-03-25

**Authors:** Pei Ma, Shuyi Li, Hui Yang, Jiqiao Yuan, Ziqian Zhang, Xuyu Li, Nan Fang, Mingbao Lin, Qi Hou

**Affiliations:** Institute of Materia Medica, Chinese Academy of Medical Sciences & Peking Union Medical College, Beijing, China

**Keywords:** ACO, mouse model, pulmonary inflammation, RNA-seq transcriptome analysis, molecular mechanisms

## Abstract

Asthma–chronic obstructive pulmonary disease (COPD) overlap (ACO) is a severe clinical syndrome characterized to describe patients with both asthma and COPD clinical characteristics, which has posed a serious threat to patients’ quality of life and life safety. However, there are many difficulties and uncertainties in its diagnosis and treatment in clinic; especially, its animal model has not been fully and thoroughly established, and the evaluation of therapeutic drugs is still in its infancy. Here, we used ovalbumin (OVA), lipopolysaccharide (LPS), and smoke costimulation to establish an ACO mouse model and then used RNA-seq technology to detect gene expression in mouse lung tissue. The results showed that ACO mice showed an overlap syndrome of asthma and COPD in lung histological changes and the levels of inflammatory cytokines in bronchoalveolar lavage fluid. The RNA-seq analysis results showed that 6,324 differentially expressed genes (DEGs) were screened between the ACO group and the control group, of which 2,717 (42.7%) were downregulated, and 3,607 (57.3%) were upregulated. Metascape analysis results showed that in the ACO model we established, due to the damage of the respiratory system, the accumulated diseased tissue involves lung, spleen, blood, bone marrow, thymus, etc. It has certain characteristics of pneumonia, pulmonary fibrosis, and chronic obstructive airway disease, lung tumors, rheumatoid arthritis. Gene Ontology and Kyoto Encyclopedia of Genes and Genomes analysis showed that DEGs were enriched in inflammation, immune system activation and imbalance, cell proliferation, and adhesion migration, and the upstream signaling pathways of inflammation were mainly affected by HLA-DRA, SYK, CTLA4, VAV1, NRAS, and JAK3. In short, our research established a mouse model that can better simulate the clinicopathological characteristics of ACO and suggested the foundations in elucidating the molecular mechanisms for pulmonary inflammation and fibrosis in ACO. This work may help further research and contribute substantially to prevention and clinical treatment of ACO in the future.

## Introduction

Asthma–chronic obstructive pulmonary disease (COPD) overlap (ACO) is a well-accepted concept defining persistent airflow limitation with features of asthma and COPD. Because of the difference in the clinical course, it is thought the pathogenesis of ACO is different from asthma or COPD. Asthma is usually characterized by airway hyperresponsiveness (AHR), leading to reversible airflow obstruction based on type 2 inflammation with eosinophils. In contrast, COPD shows progressive and irreversible airflow obstruction typically caused by smoking and associated with neutrophilic inflammation involving CD8^+^ lymphocytes and macrophages (Izuhara and Barnes, 2019). Asthma and COPD have some similarities, such as airflow obstruction, pulmonary inflammation, and AHR. Sometimes, the distinction between asthma and COPD becomes blurred, especially in asthmatic subjects who smoke or in acute exacerbations of patients with COPD (Cai et al., 2010). Compared with simple asthma and COPD, ACO episodes are more frequent, the condition is more serious, the frequency of hospitalization increases, the medical utilization rate and medical expenses increase significantly, the quality of life decreases, and the survival time is significantly shortened. Unfortunately, there are many difficulties and uncertainties in its diagnosis in clinic, and no effective treatment or clinically available pharmaceutical has hitherto been developed to resolve ACO. It has become fundamentally important to uncover the underlying mechanisms in ACO.

Systemic inflammation is a feature of chronic inflammatory airway disease, including COPD and asthma. Inflammation in COPD is typically driven by T_H_1 immune responses, whereas asthma-associated inflammatory pathways (e.g., eosinophilia and T_H_2 inflammation) appear to underlie disease in some ACO patients (Christenson et al., 2015). Studies have shown that ACO patients have significantly higher FeNO and blood eosinophil counts and percentages than COPD patients, as well as increased total and specific immunoglobulin E (IgE) levels. However, similarities between gene expression profiles in asthma and COPD have not been studied but could add to our understanding of the biology underlying the clinical and pathologic overlap between asthma and COPD. Furthermore, ACO is a complicated systematic disorder that requires explorations beyond clinical analysis, and its animal models have not yet been established. Animal studies with high-throughput transcriptome analysis are needed to comprehend the whole picture of molecular events in ACO thoroughly.

Therefore, in the present study, an ACO mouse was established, and a comparative RNA-seq strategy was recruited to evaluate the transcriptional alternations in ACO mice. The ACO mouse model was validated with histological analyses and the levels of inflammatory cytokines in bronchoalveolar lavage fluid (BALF). RNA was extracted from pulmonary tissues to construct the cDNA libraries. High-throughput sequencing was subsequently conducted on the Illumina HiSeq platform. Differential gene expression and bioinformatics analyses revealed key genes and pathways. The main altered expressions of targeted genes involved in ACO were examined by quantitative reverse transcription–polymerase chain reaction (qRT-PCR). Accordingly, the reported results from this study could lay the foundations in elucidating the molecular mechanisms for pulmonary inflammation and fibrosis in ACO and contribute substantially to its prevention and treatment.

## Materials and Methods

### Animals

In total, 80 male BALB/c mice (16–18 g) were obtained from the Vital River Laboratory Animal Technology Co., Ltd (Beijing, China). Mice were housed in an SPF environment and maintained on standard mouse chow at a temperature of 24°C ± 1°C and 12/12-h light–dark cycles, with water *ad libitum*. All experimental animal procedures were approved by the Experimental Animal Care and Use Committee of the Institute of Materia Medica, Chinese Academy of Medical Sciences & Peking Union Medical College.

### Models Building

**FIGURE 1:**
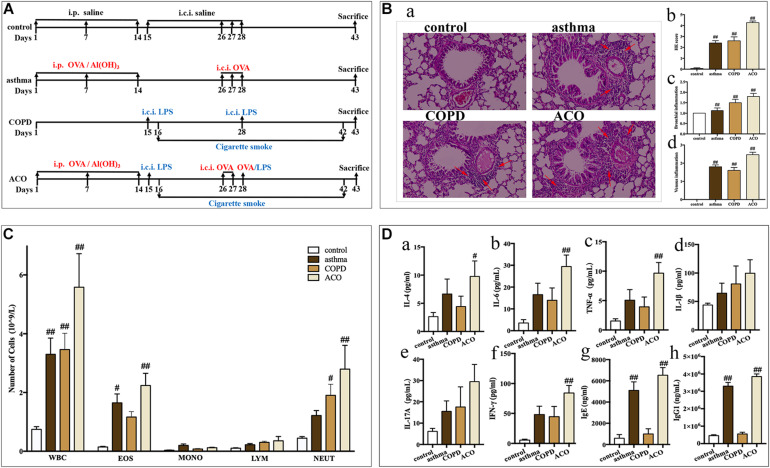
Validation of mouse ACO model. **(A)** timeline of the animal experimental protocol. **(B) (a)**: hematoxylin-eosin staining of lung tissue (magnified × 100) and its scores of inflammatory cells infiltration, **(b)**: total infiltrating scores, **(c)**: bronchial infiltrating scores, **(d)**: venous infiltrating scores (*n* = 15 independent slices from three mice of each group have been used); arrow shows inflammatory cell infiltration. **(C)** total and differential leukocyte counts in BALF (*n* = 5). **(D)** the level of IL-4 **(a)**, IL-6 **(b)**, TNF-α **(c)**, IL-1β **(d)**, IL-17A **(e)**, and IFN-γ **(f)** in BALF (*n* = 5) and the level of IgE **(g)** and IgG1 **(h)** in serum (*n* = 6). Data were shown as mean ± SEM; ^#^*P* < 0.05 and ^##^*P* < 0.01 compared with the control group.

Mice were randomly divided into four groups (*n* = 20): control, asthma, COPD, and ACO group. Mice in the asthma group were sensitized by intraperitoneal injection of 30 μg OVA and 100 μL aluminum hydroxide gel on days 1, 7, and 14, and challenged with endotracheal instillation of OVA (60 μg) on days 26–28. Mice in the COPD group received endotracheal instillation of LPS (30 μg) on days 15 and 28 and then suffered cigarette smoke for 60 min every day on days 16–27 and days 29–42. Mice in the ACO group received both OVA and LPS + smoke-inducing condition. The control group underwent an identical schedule for sham-inducing condition by saline. Animals were sacrificed on day 43 (Figure 1A).

### Pulmonary Histopathology

Mice were sacrificed 24 h after the last cigarette smoke exposure. The left lungs (*n* = 6 in each group) were excised, fixed in 10% formalin, paraffin-embedded, and sectioned at 5 μm for hematoxylin–eosin staining. Experienced pathologists gave a blinded scoring for inflammatory reaction in the lungs with a four-grade scale (Supplementary Table 1).

### Bronchoalveolar Lavage Fluid and Blood Collection

The BALF was obtained by intratracheal instillation of 800 μL 4°C phosphate-buffered saline triply and centrifuged at 1,000*g* for 10 min at 4°C. The supernatants were collected and stored at −80°C. The cells in the pellet were resuspended and quantified for the numbers of total cell, neutrophils, lymphocytes, monocytes, and eosinophils with a hemocytometer (Mindray BC-5000 vet, Shenzhen, China). Blood was collected from the retro-orbital plexus vein, clotted, and centrifuged at 1,000*g* for 10 min. The serum was stored at −80°C.

### Measurement of Inflammatory Cytokines and Immunoglobulins

The levels of interleukin 4 (IL-4), IL-6, tumor necrosis factor α (TNF-α), interferon γ (IFN-γ), IL-1β, and IL-17A in BALF supernatants and levels of total IgE, and IgG1 in serum were determined using the immunoassay kits (BioLegend, San Diego, CA, United States) according to the manufacturer’s instructions, respectively. There were five samples in each group (*n* = 5), and each sample was assayed in triplicate.

### RNA Extraction, Library Preparation, and Sequencing

Total RNA from pulmonary tissues (*n* = 3) in each group was isolated using Trizol and quantified. Sequencing libraries were generated using the NEBNext Ultra RNA Library Prep Kit (Illumina) following the manufacturer’s recommendations. The cDNA fragments between 150 and 200 bp were ligated, amplified, and purified. After cluster generation, the library was sequenced on an Illumina HiSeq X Ten System (Illumina, San Diego, CA, United States).

### Differentially Expressed Gene

Raw reads were processed through cut adapt software to remove low-quality reads or reads containing adapter and ploy-N. Reference genome and gene model annotations were accessed from GenBank. Paired-end reads were aligned to the reference genome by HISAT2 and SAMtools. Stringtie was used to count the numbers of reads mapped to each gene. Fragments per kilobase of exon per million fragments mapped of each gene were calculated based on the length of the gene and read counts mapped to this gene. Differential gene expression analysis was performed using DESeq2. The *P* values were adjusted by the Benjamini–Hochberg method. A corrected *P* value of 0.05 and log2 (fold change) of 0 were set as the threshold for differentially expressed gene (DEG).

### Bioinformatics Analysis

Correlation and principal component analysis (PCA) were performed by the corrplot and prcomp function in R 3.6, respectively. The volcano plot, Venn diagram, and heatmap were generated by ggplot2, VennDiagram, and ComplexHeatmap package in R3.6, respectively. For comparison among four groups, DEGs were input in Metascape^1^. The enrichment results in DisGeNET, PaGenBase, Reactome, TRRUST, and Hallmark were given.

The gene symbols and fold changes were input for the gene set enrichment analysis in GSEA (v4, software.broadinstitute.org/gsea/index.jsp/), with 1,000 permutations to calculate the *P* value. Gene set items with FDR < 25% and *P* < 0.05 were considered as significant. Gene Ontology (GO) analysis of DEGs was implemented by the GOseq R package. GO terms with corrected *P* < 0.05 were considered significantly enriched. The enriched DEGs in the Kyoto Encyclopedia of Genes and Genomes (KEGG)–related pathways were analyzed by the KEGG orthology–based annotation system. The significant GSEA, GO, and KEGG enrichment items were integrated using ggplot2 package in R 3.6.

A computational approach (CIBERSORT)^2^ was used in order to estimate the relative proportions of immune cell types. CIBERSORT analyses were associated with the recruitment of T cells, B cells, dendritic cells, and so on, which were done with 100 permutations. The results were filtered by setting the maximum *P* value to 0.05.

Differentially expressed genes in more than two significant GSEA items were collected and analyzed with ComplexHeatmap package in R. Their relationship was searched in STRING^3^ with medium confidence score of 0.4. Their expression levels, connection degree, and correlation were shown in the interaction network in Cytoscape 3.7.2.

### RT-PCR for Significant DEGs

Total RNA was isolated from pulmonary tissues (*n* = 3) using TRIzol (Invitrogen, Carlsbad, CA, United States) according to the manufacturer’s instructions. The first-strand cDNA was synthesized using random mixture primers and reverse transcriptase. Commercial primers were synthesized by XYbiotech (C3ar1: DMM965278, Ccr5: DMM423795, Cxcl9: DMM637393, Tnfrsf1b: DMM624034, Tnf: DMM445781, Nlrp3: DMM022617, Il17a: DMM647511, Cybb: DMM106486, Rac2: DMM036879, Nras: DMM920382, C3: DMM569106, Itgb2: DMM604973, Vav1: DMM149271, Ctla4: DMM208963, Csf2ra: DMM578340, Pik3cd: DMM102497, Il2ra: DMM956334, Jak3: DMM047007, Syk: DMM519482, Cd4: DMM869000, Pdcd1: DMM949237, H2-Ea-ps: DMM984234, Lcp2: DMM812408, Fcgr3: DMM466897, Fgr: DMM480496). qRT-PCR reactions were carried out in a real-time PCR machine (MYGO PRO, IT-IS, Ireland) with a solution (20 μL) containing SYBR Green PCR master mix (TOYOBO), cDNA (10 ng), and primers (5 pmol). Relative gene expressions were determined by comparing to the expression of the endogenous reference gene (mouse β-actin) using arithmetic formula 2^–ΔΔCT^.

### Statistics

Data are expressed as mean ± standard error of mean (SEM). Statistical analysis was performed using the SPSS software and analysis of variance. R 3.6 was used for bioinformatics analysis. Differences were considered statistically significant if *P* < 0.05.

## Results

### Validation of Mouse ACO Model

#### Animal General Observation

Compared to mice in the control group, asthmatic mice showed sneeze, cough, tachypnea, wheezing, mouth breathing, an increase of secretions, anorexia, and withered hair. COPD mice showed cough, tachypnea, opisthotonos, slackened skin, anorexia, depression, sluggishness, withered hair, and emaciation. ACO mice showed cough, tachypnea, wheezing, rales, an increase of secretions, anorexia, faded hair, and a thin and small body. All their symptoms were similar to the clinical features and disease course.

#### Lung Tissues Histological Examinations

As shown in Figure 1B, compared to the control group, the central airway of the asthma mice showed significant inflammatory changes and marked increase of infiltrating inflammatory cells (especially eosinophils) in the bronchial cavity, with concurrent bronchial submucosal edema and hyperplasia of smooth muscle. While inflammatory changes in COPD mice showed predominantly neutrophils and lymphocytes infiltrating in the bronchial cavity, with concurrent bronchial hyperplasia of smooth muscle. The histopathology of the ACO mice was more similar to that of the COPD mice, but the infiltration comprised eosinophils and made up diffuse lesions. Results of inflammatory cell infiltration scoring showed that, in ACO mice, there was more extensive infiltration of inflammatory cells into bronchi and vein regions compared to asthma and COPD mice (Figure 1Bb–d).

#### Total and Differential Leukocyte Counts and Inflammatory Cytokines in BALF

As shown in Figure 1C, compared to the control, the numbers of total leukocytes, lymphocytes, monocytes, neutrophils, and eosinophils were all increased in BALF of asthma, COPD, and ACO mice. Among these, in asthma mice, there were marked increases in the total leukocytes and eosinophils (*P* < 0.05 or 0.01), whereas marked increases of total leukocytes and neutrophils in COPD mice were observed (*P* < 0.05 or 0.01). In ACO mice, an overlap of asthma and COPD with marked increases of total leukocytes, neutrophils, and eosinophils was shown (*P* < 0.05 or 0.01).

Furthermore, an overlap in inflammatory cytokines in BALF of ACO was also shown. Compared to the asthma and COPD groups, higher levels of IL-4 (Figure 1Da), IL-6 (Figure 1Db), TNF-α (Figure 1Dc), IL-1β (Figure 1Dd), IL-17A (Figure 1De), and IFN-γ (Figure 1Df) in ACO were observed, and there were significances in IL-4, IL-6, TNF-α, and IFN-γ compared to the control group (*P* < 0.01). Additionally, the levels of total IgE (Figure 1Dg) and IgG1 (Figure 1Dh) in asthma and ACO were significantly increased, whereas those in the COPD group were almost.

### RNA-Seq Transcriptome Data of Lung Tissues

A total of 179,733; 189,911; 221,803; and 226,360 (M) raw reads were obtained from the control, asthma, COPD, and ACO groups, respectively. Following quality control, a total of 171,689; 183,66; 214,532; and 219,626 (M) clean reads were attained, respectively. Greater than 91% of the clean reads had a quality score equal to or greater than the Q30 level (sequencing error rate 0.01%), supporting the preciseness of sequencing. The data characterization was evaluated by PCA. The RNA expression profiles in the same groups were clustered together, with a positive Pearson correlation coefficients (Supplementary Figure 1A). The proportion of the total variance explained by the first and second principal components (PC1 and PC2) were 20.78 and 18.15%, respectively (Supplementary Figure 1B).

### DEG Analysis

**FIGURE 2:**
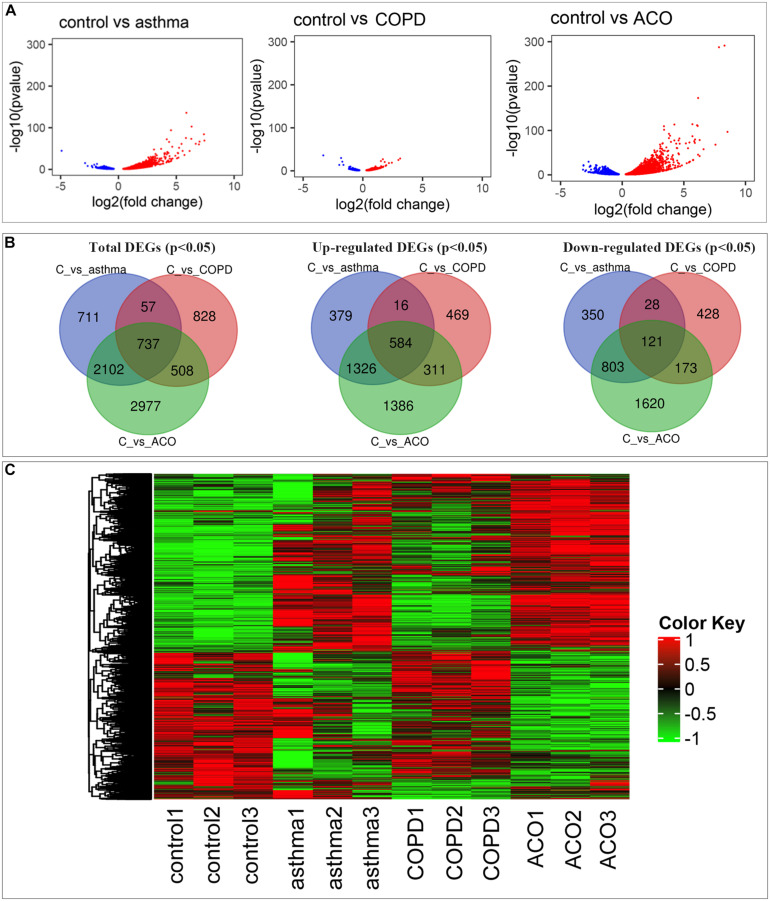
Comprehensive analysis of differential expressed genes (DEGs). **(A)** comparison of DEGs in control, asthma, COPD, and ACO groups using volcano map. Red dots represent upregulated DEGs, blue dots represent downregulated DEGs, and gray dots represent non-DEGs. **(B)** the overlap and unique DEGs in each group using Venn diagram. **(C)** the expression patterns of DEGs in different groups. DEGs were identified as *P* < 0.05 and log2 [fold change] > 1.

Compared with control, a total of 3,607; 2,130; and 6,324 genes (11.82, 6.95, and 20.12% of the total 30,526; 30,635; and 31,437 genes, respectively) were reported as significant DEGs, which comprised 2,305; 1,380; and 3,607 upregulated genes (accounting for 63.90, 64.79, and 57.04% of all significant DEGs) and 1,302; 750; and 2,717 downregulated genes (accounting for 36.10, 35.21, and 42.96% of all significant DEGs) in the asthma, COPD, and ACO groups, respectively. The detailed information of significant DEGs is shown in Figure 2 and Supplementary Table 2.

As shown in Figures 2A,C, more genes were upregulated than downregulated in pulmonary tissues of asthma, COPD, and ACO groups. Venn diagrams showed some overlapping and exclusively expressed genes in each group: 737 DEGs were involved in all groups; 2,839 overlapped DEGs were between ACO and asthma group; 1,245 overlapped DEGs were between the ACO and COPD groups; and 2,977 specific genes were expressed in the ACO group (Figure 2B). These results indicated that the gene regulation of lung inflammation induced by the two different stimuli of asthma and COPD was similar to a certain extent, but more separate properties. The overlap ratio of DEGs between the ACO and asthma groups was more significant than that between the ACO and COPD groups. However, regardless of the overlap ratio, the ratio of ACO-specific genes was more excellent. Combined with the pathological conditions, it could be concluded that the inflammatory response of ACO was more substantial, and the lung damage was more serious.

### Disease Pattern of Asthma, COPD, and ACO Mouse Model

Using Metascape to analyze DEGs, 6,446 mouse-derived genes can be identified (except for new genes and genes that cannot be annotated), and 5,775 genes homologous to humans were selected for further enrichment analysis.

**FIGURE 3:**
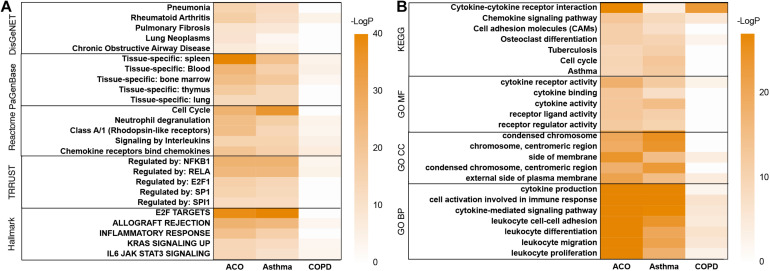
Metascape’s analysis of gene expression trends of three models. **(A)** disease pattern analyzed by using DisGeNET, PaGenBase, Reactome, TRRUST, Hallmark. **(B)** enrichment analysis and functional annotation of the selected genes performed by GO and KEGG database.

In the analysis of the DisGeNET database, the results showed that the ACO model had specific characteristics of pneumonia, pulmonary fibrosis, chronic obstructive airway neoplasms, lung tumors, and rheumatoid arthritis, indicating that the ACO model was a type of abnormal activation of immune system diseases with lung inflammation and tracheal lesions caused by allergic stimuli, which was very consistent with the clinical manifestations of ACO. PaGenBase database enrichment results showed that because of the damage of the respiratory system, the accumulated diseased tissues involved lung, spleen, blood, bone marrow, and thymus. Hallmark gene set analysis results showed that inflammation and proliferation were apparent in ACO, especially E2F targets, allograft rejection, inflammatory response, KRAS, and IL-6 signaling pathways. The TRRUST gene set indicated that the transcription factors involved were mainly the nuclear factor-kappa B (NFKB) pathway (Figure 3A).

As shown in Figure 3B, GO and KEGG database enrichment analysis and functional annotation of the selected genes were performed. The analysis results showed that these genes mainly interfered with the inflammatory process, especially cytokine interaction, lymphocyte proliferation, adhesion and migration, immune system cell activation, and abnormal neutrophil function, which were very consistent with the clinical process of ACO.

### Potential Signaling Involving in the Asthma, COPD, and ACO Model

**FIGURE 4:**
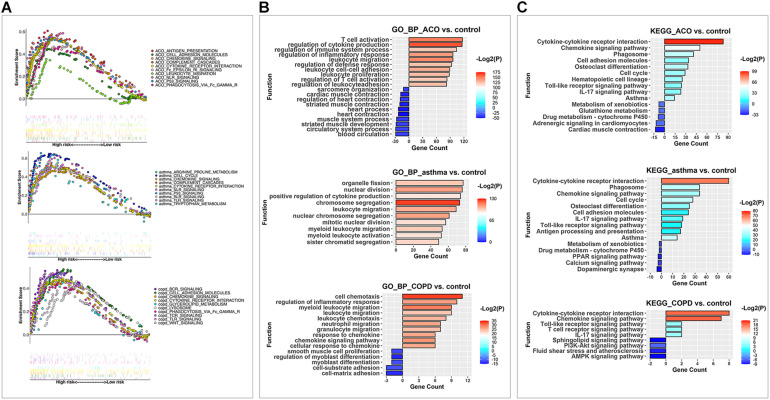
GSEA, KEGG, and GO analysis were used to analyze the key pathways of DEGs in different models. **(A)** the enriched KEGG pathways in each model after GSEA analysis. Each point represented a gene; the scattered points in upward trend indicated positive contributions to the enrichment of this item, and that in downward trend demonstrated negative contributions. Each vertical line represented the gene that contributes to this item. Different colors represented different items, and the straight line connected end to end expressed the same gene in additional items. **(B)** the enriched upregulated and downregulated biological process items using GO BP analysis. **(C)** the enriched upregulated and downregulated pathways by KEGG analysis.

Gene set enrichment analysis was performed on 7,920 DEGs. The group type was taken as the hypothesis test type and tested 1,000 times, and the number of genes in the test gene set was 15–500. The top 20 abundant terms with a nominal *P* value < 1% in the significantly enriched gene set were chosen to illuminate the functions of the DEGs further. We further used the R package ggplot2 to compare multiple GSEA entries (Figure 4A). To find out the critical gene groups that can regulate multiple items, we analyzed the genes in the pathway, collected genes that appeared in at least two things, and performed heatmap analysis on these genes. We focused on the genes that were significantly increased in the ACO group and their related DEGs. The results showed that the ACO group was enhanced considerably in antigen presentation, cell adhesion molecules, chemokine signaling, cytokine receptor interaction, complement cascades, Fcε and Fcγ pathways, and NLR and P53 signaling pathway. The asthma group was enriched in the cell cycle; cytokine and cytokine receptor interaction; complement cascades; NLR, P53, RLR, TLR signal pathway; and arginine and tryptophan metabolic pathway. The COPD group was significantly enriched in BCR, TCR, TLR, WNT signaling pathway, Fcγ, cell adhesion molecules, cytokines, cytokine receptor interaction, glyceride metabolism, lysosomes, and other ways. This showed that the ACO group not only reflected the high expression of cytokines and the interaction between cytokines and receptors in asthma and COPD groups, but also had the characteristics of NLR and P53 of asthma, the cell adhesion, and Fcγ characteristics of COPD and specifically activate antigen presentation, cell migration, and Fcε pathway.

And then, up and down DEGs were separately analyzed by GO (Figure 4B) and KEGG (Figure 4C) analysis. Results of the GO analysis showed that the DEGs were concentrated in inflammation, immune system activation and imbalance, and cell proliferation, adhesion, and migration. The most significantly enriched category was the “regulation of cytokine production.” Other types related to interactions and responses of immune cells (leukocytes, lymphocytes, and T cells) were also predominately enriched. Based on these, the DEGs in different functional categories may provide valuable information for future evaluations of ACO. Furthermore, in the KEGG analysis, the biochemical pathways of the DEGs were investigated. Among these subcategories, the most significantly enriched way was cytokine–cytokine receptor interaction. Other enriched pathways considerably included chemokine signaling pathway, phagosome, and cell adhesion molecules (corrected *P* < 0.05). Therefore, our subsequent analysis focused on inflammation caused by abnormal lymphocyte proliferation and activation in ACO.

### Immune Cell Type and Content Analysis

**FIGURE 5:**
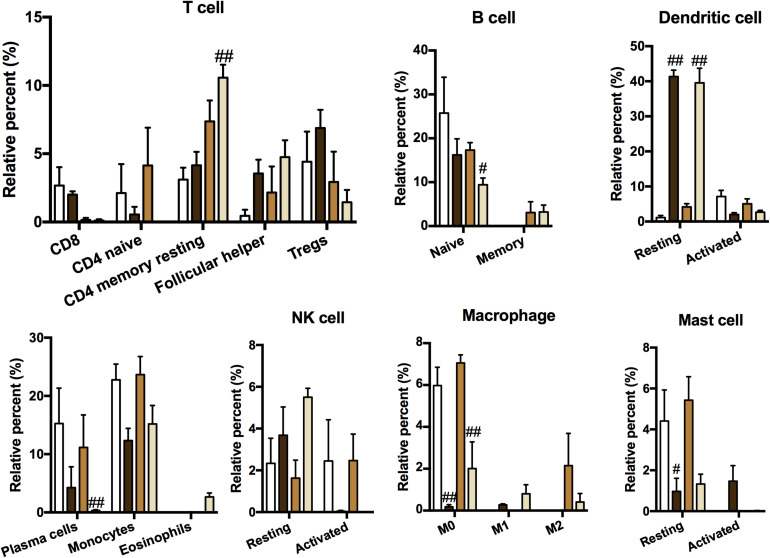
Immune cell type and content analysis. The immune cell subtypes and relative expression levels were reconstructed according to the expression pattern of cell surface markers at the RNA level using CIBERSORT (*n* = 3). Data were shown as mean ± SEM; ^#^*P* < 0.05 and ^##^*P* < 0.01 compared with the control group.

Because of the enhanced immune response in ACO mice, the relative abundances of 22 immune cells were calculated by using the CIBERSORT algorithm (an algorithm based on linear support vector regression to deconvolute the expression matrix of immune cell subtypes reconstructs immune cell subtypes and immune cell infiltration conditions based on the expression patterns of cell surface markers at the RNA level). As shown in Figure 5, plotting 19 kinds of cells with abnormal expression showed that T cells, B cells, macrophages, and other cells were activated in different proportions in the asthma, COPD, and ACO models. Recruitment of CD4^+^ T cells and eosinophils was abnormally higher in ACO mice, indicating a stronger tendency of asthma, whereas B cells, macrophages, and dendritic cells showed more robust activation.

### Core Genes in Inflammation Signaling

**FIGURE 6:**
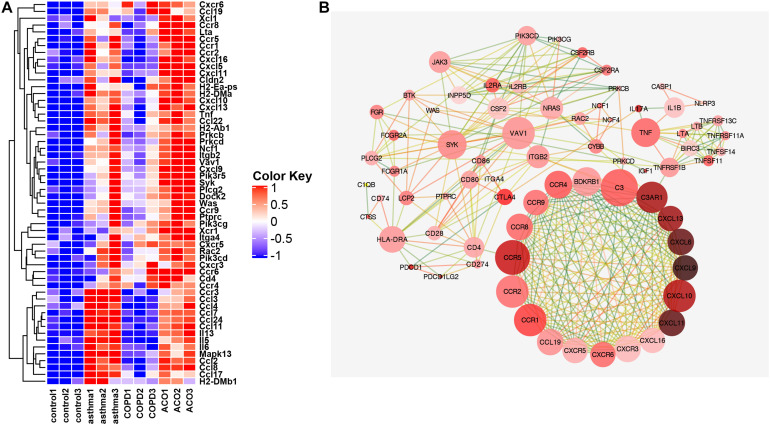
Key genes of inflammation pathway. **(A)** the FPKM expression levels of genes appearing in more than two pathways in GSEA analysis are shown. **(B)** protein interaction network diagram of genes with correlation coefficient >0.9. The color of dot display (light red to dark red indicate that the expression multiple has increased from 0.49 to 5.7 times) indicated the multiple difference between the ACO and control group [log2 (FPKM) value]. The line represented the correlation coefficient between proteins calculated based on a string (correlation coefficient 0.9 is dark green, one is orange-red, and the correlation between 0.9 and 1 is excessive from green to orange; the closer correlation is to 1, the more vital the protein interaction is).

As shown in Figure 6, the interaction between adhesion chemokines was powerful, and their expression was very high. CD4, PRKCD, and ITGB2 mainly regulated them through CCR5 and C3. The expression of inflammatory factors TNF, IL-1, and IL-17 was high, mostly held by BIRC3, NLRP3, and CTLA4. The upstream signaling pathway of inflammation was controlled primarily by HLA-DRA, SYK, CTLA4, VAV1, NRAS, and JAK3.

### Verification of Gene Expressions via RT-PCR

**FIGURE 7:**
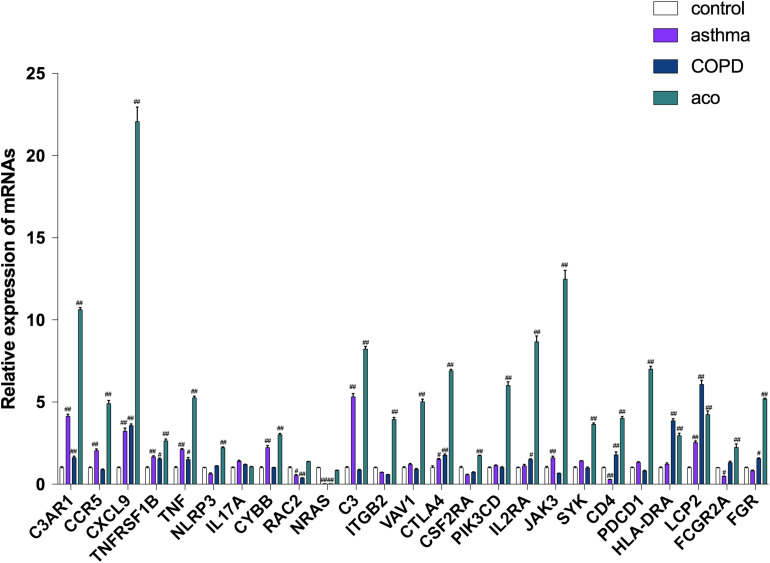
Verification of key genes by using RT-PCR. Data were shown as mean ± SEM of three independent experiments. ^#^*P* < 0.05 and ^##^*P* < 0.01 compared with the control group.

To validate the data from RNA-seq, high potential therapeutic targets that were representatives of DEGs were selected for RT-PCR analysis. As shown in Figure 7, 14 major regulatory DEGs were selected for qRT-PCR verification, including CCR5, C3, CD4, ITGB2, TNFRSF1B, TNF, IL-17, NLRP3, CTLA4, HLA-DRA, SYK, VAV1, NRAS, and JAK3. All of the 14 DEGs exhibited the same tendency between the RNA-seq analysis and qRT-PCR results, which suggested that our transcriptome analysis was accurate and reliable, and the 14 selected DEGs may be high potential therapeutic targets of ACO.

## Discussion

Asthma and COPD are two common respiratory diseases, but it is often difficult to distinguish them clearly in clinical practice, and in some patients, both diseases can coexist. To date, most clinical studies of respiratory diseases have included only patients who clearly meet the diagnostic criteria of asthma or COPD (Papaiwannou et al., 2014). The treatment guidelines also lack management advice for patients who suffered from both asthma and COPD. With the growing awareness of the prevalence of asthma and COPD coexistence, more attention has been focused on meeting the needs of patients with ACO. However, there are many difficulties and uncertainties that hinder further deepening research on ACO; especially, its animal model has not been fully and thoroughly established, and the evaluation of therapeutic drugs is still in its infancy. Therefore, establishing a reliable ACO animal model that meets clinical characteristics has become a top priority.

The clinical manifestations of ACO are similar to asthma and COPD. The main symptoms are chronic cough, wheezing, post-exercise shortness (Bleecker, 2004), allergic rhinitis, anxiety, and osteoporosis (van Boven et al., 2016). The release of T_H_1 cytokine (IFN-γ) was reduced in ACO patients, and the production of T_H_2 cytokines (IL-4) and IgE increased, indicating that ACO patients converted to a T_H_2 immune response advantage (Kalinina et al., 2016). The presence of both sputum eosinophils and sputum neutrophils is increased in ACO (Iwamoto et al., 2014); especially, eosinophils play an important role in this (Bobolea and Llano, 2016). In terms of pathology, ACO patients have significantly increased bronchial wall thickening and increased airway remodeling compared with asthma and COPD patients (Backman et al., 1997; Fujimoto et al., 2012). Several potential ways that can lead to ACO were that patients’ early-onset asthma exposure to cigarette smoke leads to the formation of fixed airflow limitation and the development of COPD (Bobolea and Llano, 2016), or that patients with COPD showing signs of late-onset asthma in later years (Bobolea and Llano, 2016). In this study, according to the clinical manifestations and possible causes of ACO, on the basis of the methods of asthma and COPD airway inflammation mouse models established, an ACO mouse model was established by using OVA, LPS, and smoke costimulation. The results showed that symptoms of ACO mice were similar to their clinical features and disease course. The histopathology of ACO mice was more similar to that of the COPD mice, but the infiltration comprised eosinophils, and there was more extensive infiltration of inflammatory cells into bronchi and vein regions compared to asthma and COPD mice. In ACO mice, there was an overlap of asthma and COPD with marked increases of total leukocytes, neutrophils, and eosinophils counts and higher levels of inflammatory cytokines (IL-4, IL-6, TNF-α, IL-1β, IL-17A, and IFN-γ) in BALF. Additionally, serum IgE and IgG1 in asthma and ACO were significantly increased, whereas those almost unchanged in COPD mice. These results indicated that the ACO mouse model established in this study was shown to meet clinical ACO characteristics, which may be a potential reliable ACO animal model for deepening ACO research.

RNA-seq technology has become a new and more effective method for large-scale research on the transcriptome. For its high resolution, accuracy, and high data throughput, RNA-seq technology has broken through the limitations of previous technologies and is widely used in genome, epigenome, exome, and transcriptome sequencing. Especially, it has obvious advantages in the study of potential mechanisms and targets of complex diseases, to have a more comprehensive understanding of its entire transcriptome. More importantly, RNA-seq can directly recognize RNA sequence files, which is essential in analyzing unknown genes and new transcript isoforms (Marioni et al., 2008; Wilhelm et al., 2008). Here, as there are many uncertainties and complexities in ACO diagnosis and treatment in clinic, RNA-seq technology was used to detect gene expression in ACO mouse lung tissue to elucidate the potential molecular mechanisms and targets for pulmonary inflammation and fibrosis in ACO mice. The results showed that 6,324 DEGs were screened between the ACO group and the control group, of which 2,717 (42.7%) were downregulated, and 3,607 (57.3%) were upregulated. Metascape analysis showed certain characteristics of pneumonia, pulmonary fibrosis, and chronic obstructive airway disease, lung tumors, and rheumatoid arthritis in the ACO mice. And by using GO and KEGG analyses, DEGs were enriched in inflammation, immune system activation and imbalance, and cell proliferation and adhesion migration, and the upstream signaling pathways of inflammation were mainly affected by HLA-DRA, SYK, CTLA4, VAV1, NRAS, and JAK3.

In this study, a set of airway epithelial genes that are altered in the mice model of asthma and COPD suggests similar processes leading to airflow obstruction in ACO. Pathway analysis suggests the innate immune pathways of cell adhesion and chemokine signaling, introduced by CXCLs (6,9,10,11,13), CXCRs (3,5,6), and CCRs (1,2,4,5,8,9), which are activated by upstream mediators including NLRP3 and subsequently IL-1 and TNF, or activated by JAK, Syk, and subsequently CD4. Through qRT-PCR verification that CXCL9 expresses the highest in the ACO model compared with the control among these DEGs, this finding suggests that CXCL9 may be a biomarker of autoimmune inflammation in patients with ACO. The modulation by Syk linker B phosphorylation of the Syk-Vav SH2-binding affinity is a primary element of regulation in B-cell signaling (Chen et al., 2011), which activated both ERK2 and JNK (Miranti et al., 1998). In addition, Vav1 phosphorylation can efficiently cooperate with T-cell receptor signaling to enhance NFAT-dependent and NFKB-dependent transcription, which requires Syk as it is expressed in all hematopoietic cell types, suggesting Syk may allow integrins to couple with Vav in hematopoietic cells. And Vav plays a critical role in linking FcepsilonRI and Syk to the Rac1-JNK pathway in basophils, mast cells, T cells, and B cells (Teramoto et al., 1997). This further suggests that the gene expression changes of ACO reflect biology beyond T_H_2 inflammation. Thus, it is undeniable that ACO has clinical universality of asthma and COPD.

In conclusion, ACO has posed a serious threat to patients’ quality of life and life safety, reminding us that ACO should be given enough attention. In this study, a mouse model was established, which can better simulate the clinicopathological characteristics of ACO. That inflammation, immune system activation and imbalance, and cell proliferation and adhesion migration were mainly affected by HLA-DRA, SYK, CTLA4, VAV1, NRAS, and JAK3 signaling pathways was suggested to be the potential molecular mechanism for pulmonary inflammation and fibrosis in ACO. However, there are many limitations in this study; future research on ACO still requires a large number of animal experiments to provide evidences.

## Data Availability Statement

The datasets presented in this study can be found in online repositories. The names of the repository/repositories and accession number(s) can be found in the article/Supplementary Material.

## Ethics Statement

The animal study was reviewed and approved by the Experimental Animal Center of the Institute of Materia Medica, Chinese Academy of Medical Sciences & Peking Union Medical College (Beijing, China).

## Author Contributions

SL performed the animal experiments and analyzed the data. PM performed RNA-seq analysis and analyzed the data. PM and SL wrote portions of the manuscript draft. HY, JY, ZZ, XL, and NF participated in some experiments and data analysis. ML and QH designed the experiments, analyzed the data, and revised the manuscript. ML oversaw the overall execution of the projects and gave final approval of the version to be submitted. All authors contributed to the article and approved the submitted version.

## Conflict of Interest

The authors declare that the research was conducted in the absence of any commercial or financial relationships that could be construed as a potential conflict of interest.

## Footnotes

1 https://metascape.org

2 https://cibersort.stanford.edu/

3 https://string-db.org

## Supplementary Material

Supplementary Figure 1 The overall quality of RNA-seq transcriptome data of lung tissues. **(A)** Correlation within and between groups of RNA-seq data, calculated by correlation coefficients. **(B)** PCA database on the first three principal components.
