# Supplementary material for: Comparative RNA-Seq Transcriptome Analysis on Pulmonary Inflammation in a Mouse Model of Asthma–COPD Overlap Syndrome
Source: Front Cell Dev Biol. 2021 Mar 25;9:628957. doi: 10.3389/fcell.2021.628957 (PMC8044804; doi:10.3389/fcell.2021.628957)
Supplement: Supplementary file 1 [file Table_1.DOCX]

Table S1 Bronchi-perivascular inflammation score description.

| Score | Standard for evaluation |
| --- | --- |
| 0 | No inflammatory cell infiltration. |
| 1 | Occasionally, cuff-like inflammatory cell infiltration in veins and parabronchial tubes is observed. |
| 2 | Most venous and parabronchial inflammatory cells infiltrate obviously, with 1-5 layers of inflammatory cells. |
| 3 | A large number of inflammatory cells infiltrate most veins and bronchi with more than 5 layers. |

Infiltration of inflammatory cells in veins with diameters of 100-200um and small trachea of 150-300um. Choose 5 points for each slice for scoring.
