# Supplementary figures and images for: Comparative RNA-Seq Transcriptome Analysis on Pulmonary Inflammation in a Mouse Model of Asthma–COPD Overlap Syndrome
Source: Front Cell Dev Biol. 2021 Mar 25;9:628957. doi: 10.3389/fcell.2021.628957 (PMC8044804; doi:10.3389/fcell.2021.628957)

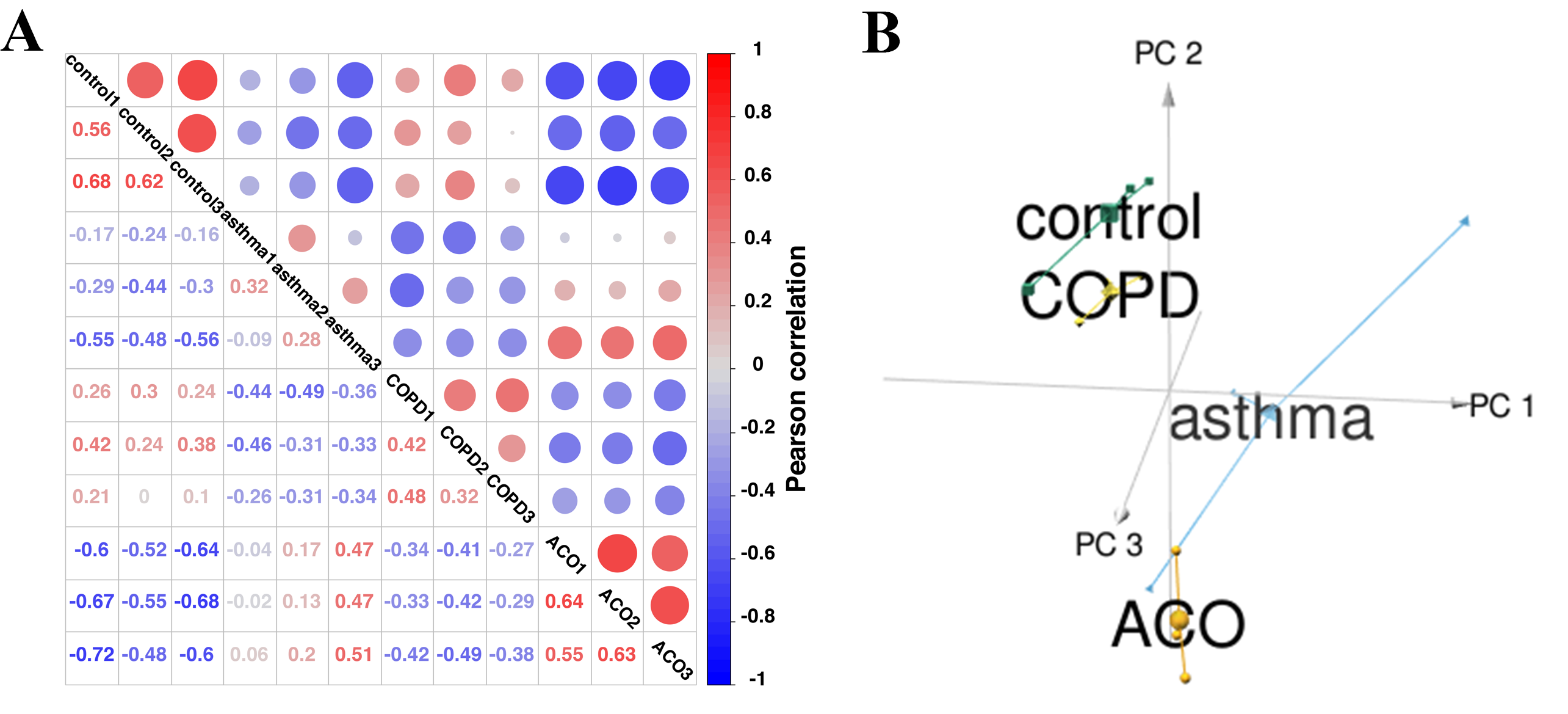

Supplement: Supplementary Figure 1 — The overall quality of RNA-seq transcriptome data of lung tissues. (A) Correlation within and between groups of RNA-seq data, calculated by correlation coefficients. (B) PCA database on the first three principal components. [file Image_1.TIF]
